# Transient Ischaemic Attack 999 Emergency Referral (TIER): a cluster randomised feasibility trial facilitated by data linkage

**DOI:** 10.23889/ijpds.v1i1.347

**Published:** 2017-04-19

**Authors:** Anne Seagrove, Jenna Bulger, Helen Snooks, Nigel Rees, Martin Heaven

**Affiliations:** 1 Patient and Population Health and Informatics, Swansea University Medical School; 2 Weslh Ambulance Services NHS Trust; 3 Farr Institute, Swansea University

## Objectives

Studies demonstrate TIA patients are at risk of further TIAs, stroke and death. TIA incidence is unknown, however estimated at 35 per 100,000 people annually in the UK, costing approximately £7 billion. Many TIA patients call 999, are assessed, stabilised and conveyed to Emergency Department (ED). Rapid assessment of TIA severity and risk and intervention is emerging as the new standard for TIA care leading to alternative pathways with direct referral to specialist services. However, uncertainties exist over this new model of care.

We will develop and assess feasibility of paramedic assessment and referral of low-risk TIA patients directly to TIA clinic for early review, thus providing timely specialist review without: adverse consequences; inconvenience of ED attendance; unnecessary cost to the NHS.

## Methods

This feasibility trial is designed to test the methods of a pragmatic cluster randomised trial, utilising data linkage for capturing outcome data, but with a qualitative component. To develop the treatment protocol, training and referral processes, working with clinicians/stakeholders, we will conduct:

- survey across UK ambulance services to find referral pathways for low-risk TIA patients
- systematic review of TIA prehospital care
- paramedic focus groups pre-implementation

Then:

- randomise paramedics (intervention/control)
- recruit patients
- interview patients, key clinicians and service managers
- collect routine data via data linkage using the SAIL databank
- hold paramedic focus groups post-implementation

We will:

- measure uptake and compliance with treatment protocols
- validate TIA assessment tool
- analyse qualitative data
- pilot recruitment processes
- test data collection methods
- estimate key outcomes effect size to inform full trial sample size calculation

## Results

Will inform full trial development using criteria: intervention acceptability to practitioners and patients; trial design feasibility; outcome data completeness.

## Conclusion

- If indicated, full trial conducted
- If not, but positive results - advise intervention development for immediate implementation
- If not, but negative results - advise delivery of intervention should cease.

